# Enhancing the primary care pediatrician's role in managing psychosocial issues: a cross sectional study of pediatricians and parents in Israel

**DOI:** 10.1186/s13584-022-00537-6

**Published:** 2022-08-04

**Authors:** Hava Gadassi, Inbal Millo David, Maya Yaari, Eitan Kerem, Manuel Katz, Basil Porter, Chen Stein-Zamir, Zachi Grossman

**Affiliations:** 1 Haruv Campus, Mt Scopus, Jerusalem, Israel; 2 Hadassah Medical Organization, Kalman Mann, Jerusalem, Israel; 3 Meuhedet Healthcare Services, Tel Aviv, Israel; 4 Maccabi Healthcare Services, Tel Aviv, Israel; 5 The Hebrew University, Hadassah Braun School of Public Health and Community Medicine, Faculty of Medicine, The Hebrew University of Jerusalem, Jerusalem, Israel; 6 Ministry of Health, Jerusalem, Israel; 7 Adelson School of Medicine, Ariel University, Ariel, Israel

**Keywords:** Pediatrics, New morbidity, Psychosocial problems

## Abstract

**Background:**

Psychosocial issues are an integral part of children's health and well-being, and it is widely acknowledged that pediatricians should be involved in their management. We examined the current perception of the pediatrician’s role in the management of psychosocial problems in Israel from the perspective of parents and pediatricians, and identified possible barriers.

**Methods:**

We assessed parents' and pediatricians' perspectives through a cross sectional survey. 1000 parents with children under 10 were randomly selected from a large database representing the Israeli population and phone-surveyed by a polling company. Due to a low response-rate (5.4%), there was an overrepresentation of married parents and underrepresentation of parents with primary or secondary education. 173 Pediatricians were recruited both at a medical conference and by a web-based questionnaire.

**Results:**

55% of the parents reported they were concerned with at least one psychosocial problem, yet less than 50% of them discussed these issues with the pediatrician. 59.9% of the parents did not perceive psychosocial problems as relevant to the pediatrician's role. Pediatricians with some previous training related to psychosocial issues were more likely to report on a lack of professional confidence (*p* = .037) and insufficient available resources (*p* = .022) as barriers to their involvement, while pediatricians who had no training were more likely to report on the parents' perception of their role as the barrier to involvement (*p* = .035).

**Conclusions:**

Parents tend to avoid the discussion of psychosocial concerns in pediatric settings due to their perception that it's irrelevant to the pediatrician's role. Trained pediatricians feel unconfident in their ability to manage psychosocial issues and report on a lack of suitable resources. These findings suggest current pediatric mental-health training is insufficient to equip pediatricians with the knowledge and skills required to their involvement in psychosocial problems, and imply necessary changes to environment of community-based pediatrics. In order to change the practice of pediatricians in the community to enable them to address a variety of psychological issues, appropriate training is needed, through all stages of the pediatrician’s professional life, including medical school, pediatric residency and continuous medical education.

**Supplementary Information:**

The online version contains supplementary material available at 10.1186/s13584-022-00537-6.

## Background

In 1995, Robert Haggerty published his vision for the future of pediatrics [1]. Haggerty claimed that pediatric practice must step into the field of “new morbidities,” including the prevention, early detection, and management of psychosocial problems. Psychosocial problems include a wide range of emotional, behavioral, and social issues, such as eating and sleeping disorders, anxiety, depression, and more. These are an integral part of children's health and well-being, and are crucial to their ability to achieve optimal physical, mental, and social functioning. A 1992 study demonstrated that these problems account for approximately 20% of problems experienced by children aged 8–15. [2]

In accordance with Haggerty's vision, guidelines for diagnosing and treating psychosocial problems were published by the *American Academy of Pediatrics* (AAP), to highlight the important role of pediatricians in children's mental health care [3, 4]. The primary care pediatrician serves at the frontline as a universal service and has access to the general population, with no stigma attached, and an ongoing relationships with families. Recent studies demonstrated that between 15 and 50% of children seen by the primary care pediatrician had significant psychosocial problems [5, 6]. About 75% of Israeli adolescents with psychosocial problems who did not consult any mental health specialist, did visit the primary care pediatrician [7]. The pediatrician is in an optimal position for early detection of psychosocial problems through routine check-ups, as well as for tracking progress and coordinating long-term care. Therefore, pediatricians should offer services such as early detection, counseling, coordination of care, and participation in a multidisciplinary team to meet children's psychosocial needs.

A variety of models in the last decade have attempted to integrate mental health care in the primary care setting [8]. One strategy includes the embedding of mental health screening tools during regular visits to the pediatrician, thus underlining the importance of training in early detection [9, 10]. A second strategy emphasizes the establishment of collaborative relationships with mental health specialists, as part of the primary care team or by frequent consultations [11, 12]. A meta-analysis in 2015 found that primary pediatric care that incorporated components of professional behavioral consultation was more efficient than regular medical and pharmaceutical care for children and teens with behavioral or emotional problems [13]. These findings support the assumption that multidisciplinary teams and psychosocial awareness create an efficient model of preliminary support at the primary level.

Although the movement towards integration of mental health in primary care setting has existed for decades, a previous study revealed that Israeli pediatricians were less likely to take an active role in managing these problems [14]. Out of 1 million pediatric contacts, less than 1% involved new morbidity issues. It further indicated that when pediatricians regarded the problem as part of their role, they were more likely to manage it by themselves or with the help of other professionals. Another study examined the role of physicians in Maternal Child Health Clinics (MCHC) as perceived by parents in Israel, and found it was characterized by a lack of uniformity, particularly regarding new morbidity issues [15]. Most parents did not see counseling and guidance as a core component of the pediatric role. The researchers suggested that this may reflect the current hospital-based training of Israeli pediatricians, and their lack of exposure to developmental and behavioral pediatrics. A recent study showed that the hospital-based training of pediatricians does not provide the necessary opportunities to learn elements of the “New Morbidity”, resulting in many community-based pediatricians choosing not to confront these problems [16]. Although residency training is essential to equip pediatricians with the knowledge and skills they need to deal with psychosocial issues, this part of their medical education varies immensely due to a lack of formal demands and supervision.

### Study goals

The study aim was to assess the current perception of the pediatrician’s role in the management of psychosocial problems in Israel from the perspective of both parents and pediatricians, and to evaluate the existing barriers to incorporating psychosocial support by pediatricians in community primary care settings. The study also aimed at examining the association between training in developmental and behavioral pediatrics and the pediatricians' involvement, and barriers regarding psychosocial issues.

## Methods

### Study design

The study was conceived as a cross sectional survey among parents and pediatricians.

### Participants

***Parents' survey***: The study included 1000 parents of children up to the age of 10, of whom 800 were Jewish, and 200 were Arabs, which represents the proportion of each ethnicity in the Israeli population. The parents' survey was conducted by the Geocartography Knowledge Group. The sample of parents was randomly selected by a Computer-assisted telephone interviewing (CATI) program from a large database, containing approximately 43,500 listings of Jews and Arabs, according to a set quota for sex, parental age, children’s age, religion, and geographical area, representing the Israeli population. Computer aided telephonic interviews were conducted with parents who provided consent. At the beginning of the survey parents were asked a screening question regarding their children's age. Only parents with children under 10 were further interviewed and included in the sample. The final response rate was approximately 5.4%. The maximum margin of error was ± 3.1%, with a 95% significance level. Due to the low response rate, representativeness of the parents' sample was examined using data gathered by the central Bureau of Statistics through the 2019 wave of the Long-term Survey of Households [17]. A comparison with the general population of parents in Israel revealed that the sample's marital status and level of education differed significantly, with overrepresentation of married parents and underrepresentation of parents with primary or secondary education (Please see Additional file 1: Table S1). ***Pediatricians' survey:*** Out of 190 community pediatricians who initially agreed to participate in the survey, a total of 173 pediatricians completed the questionnaire (91%). The representativeness of the responding physicians was evaluated by comparing characteristics of the study participants (age, gender, years in practice) with those of Israeli pediatricians as published in the Israel Ministry of Health (IMOH) report on health professionals in 2019 [18]. No significant differences were found.

The pediatricians were recruited on two occasions: during the conference of the Israel Ambulatory Pediatrics Association (IAPA) held in March 2019 (49.7% of the participants), and via the Israel Pediatric Research in Office setting NETwork (IPRONET; 50.3% of the participants) [19]. Conference attendees were recruited after the study was presented to the general assembly. All participants received a text message with a link to an online questionnaire. Printed copies were provided on request. Additional pediatricians were recruited through an online questionnaire distributed via the IPRONET mailing list used by Israeli pediatricians. The Israel Pediatric Research in Office Setting (IPROS) network was established in 1995 and includes Israeli pediatricians willing to collaborate with the network on performing research in their clinics. To avoid duplication, participants were asked for the last four digits of their ID numbers before completing the questionnaire.

### Research instruments

*Parent questionnaire* (“Appendix A”): The parent questionnaire contained relevant questions from the Promoting Healthy Development Survey (PHDS) [20], which was designed to evaluate pediatricians' diagnoses and treatment of children's psychosocial problems. The parents were asked to: 1. Provide information regarding the demographic characteristics of the family; 2. Describe their existing concerns regarding a child’s psychosocial problems (including sleep, feeding and eating, emotional aspects of medical conditions, emotional, behavioral, or social functioning problems, socioeconomic difficulties and family problems that might affect the child's health). For each subject, parents were asked to rate on a 3-point scale whether this issue did not bother them at all, somewhat bothered them, or extremely bothered them. 3. Reply whether these concerns were discussed with the pediatrician (yes/no) and who initiated the discussion (the parents/ the pediatrician); 4. Describe the care the pediatrician provided for psychosocial problems that were discussed with him: Did the pediatrician offer treatment himself, such as consultation, pharmacological care or other interventions (yes/no)? Did he refer to other services and specialists? 5. Rate the level of satisfaction with the pediatrician's care on a 1–5 Likert-scale, and 6. Choose from a defined list which barriers prevented them from discussing these problems with the pediatrician (multiple answers were accepted). The survey was pilot tested on a subset of participants and corrected according to their review.

*Pediatrician questionnaire* (“Appendix B”): The pediatrician questionnaire was mostly based on relevant questions from the AAP’s periodical survey questionnaire for pediatricians, designed to examine primary care pediatricians’ involvement in the mental health issues of their patients. Pediatricians were asked to report on: 1. Demographic characteristics, years in practice, and exposure to relevant fields during training or continuous medical education (including either a mental health rotation during hospital-training, community pediatrics or child development clinics rotation, or any other continuing education in mental health); 2. Their current practice regarding children’s psychosocial problems (sleep, feeding and eating; emotional aspects of medical conditions; emotional, behavioral, or social functioning problems; socioeconomic disparities; and family problems that might affect a child's health). For each subject, the pediatricians were asked to rate on a 1–3 scale whether they never/rarely, sometimes, or rather often: (a) initiated a discussion regarding this issue, (b) offered treatment regarding this issue themselves (such as consultation, pharmacological care, or other interventions), (c) referred to other specialists. 3. Pediatricians were asked to list which specialists did they refer to in the last year 4. Finally, pediatricians were asked to rate on 1–5 Likert-scale possible barriers to managing psychosocial problems as part of primary care. The questionnaire was validated through expert reviews.

Three practice variables were calculated by averaging all the relevant points, namely: interest in psychosocial problems, treatment of psychosocial problems, and referral to other professionals. This was possible due to a high internal consistency; the Cronbach’s alpha values were as follows: the interest variable (α = 0.88), the treatment variable (α = 0.84), and the referral variable (α = 0.80).

### Statistics

Descriptive statistics of the overall responses of the parents' and pediatricians' surveys are presented first. The correlations between the subgroups of barriers and the pediatricians' practice in psychosocial problems were examined using Pearson correlation coefficients. Finally, *t*-tests were conducted to investigate the differences in practice regarding children's psychosocial issues between pediatricians who had had further training in relevant fields and those who had not had such training. All analyses were performed with SPSS software ver. 23.0 (IBM, Armonk, NY, USA).

## Results

### Parents' survey

The participating parents’ (n = 1000) demographic characteristics are presented in Table 1.

**Table 1:** Demographic characteristics—parents

Variable	Total N = 1000
Sex (%)	
Male	37.9%
Female	62.1%
Age (Mean (SD), Range)	41 (5.9), 21–60
Marital status (%)	
Single	0.5%
Married/relationship	96.7%
Divorced	2.4%
Widowed	0.4%
Education (%)	
Primary or secondary	22.6%
Professional diploma	21.2%
Bachelor’s degree	28.8%
Masters or PhD	20.5%
Religion (%)	
Jewish	78.9%
Muslim	17.9%
Christian	1.5%
Other	1.5%
Level of religiosity	
Secular	47.1%
Traditional	8.9%
Orthodox	17.5%
Ultra-orthodox	26.5%

#### Parents’ concerns regarding psychosocial problems

**Fig. 1:**
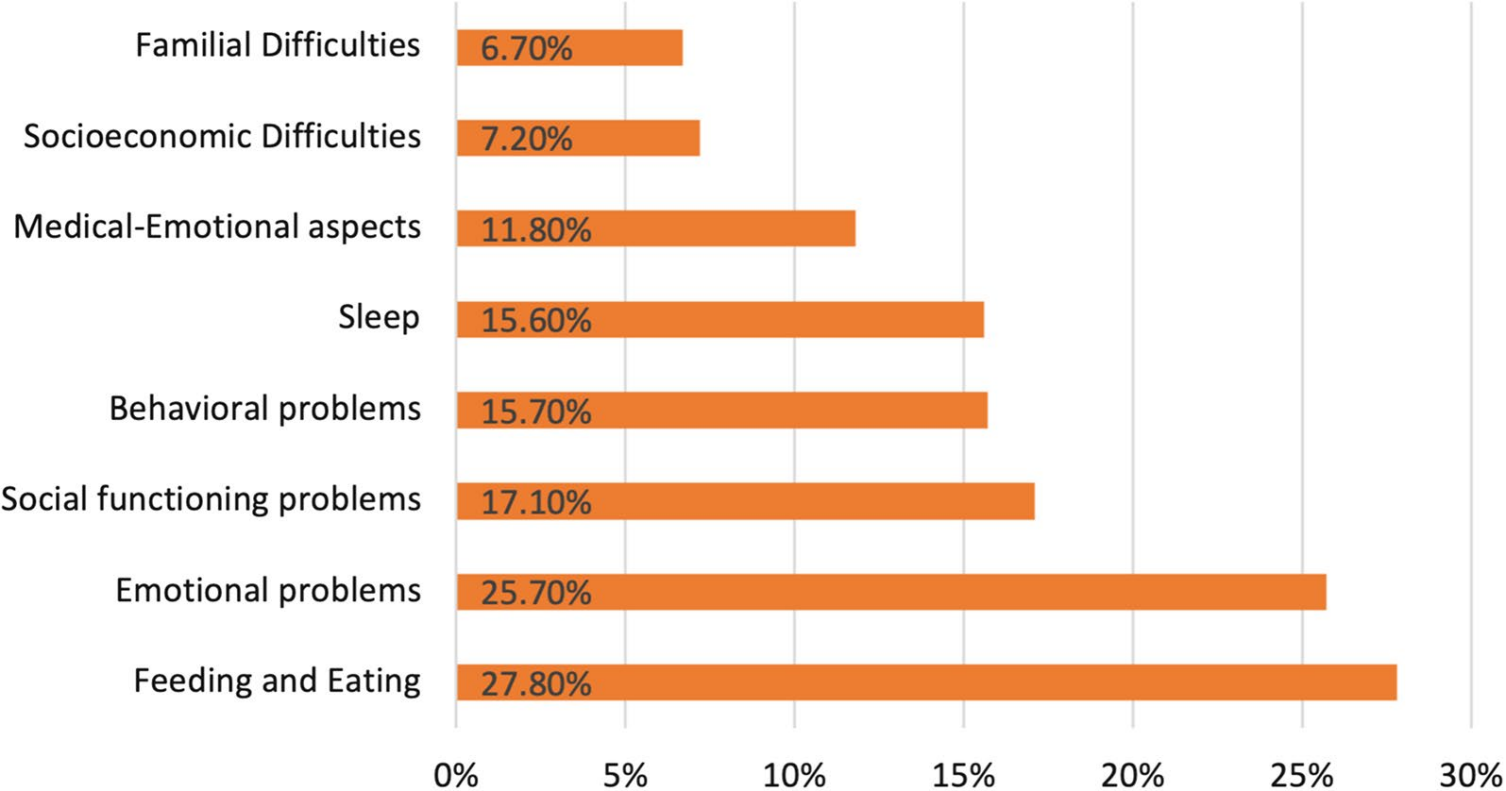
Parents’ psychosocial concerns

Figure 1 shows the parents’ concerns regarding different psychosocial problems. Roughly 55% of the responding parents had concerns regarding at least one of the issues listed. Feeding and eating and emotional difficulties were among the most prevalent of these concerns.

#### Discussing psychosocial problems with the pediatrician

**Fig. 2:**
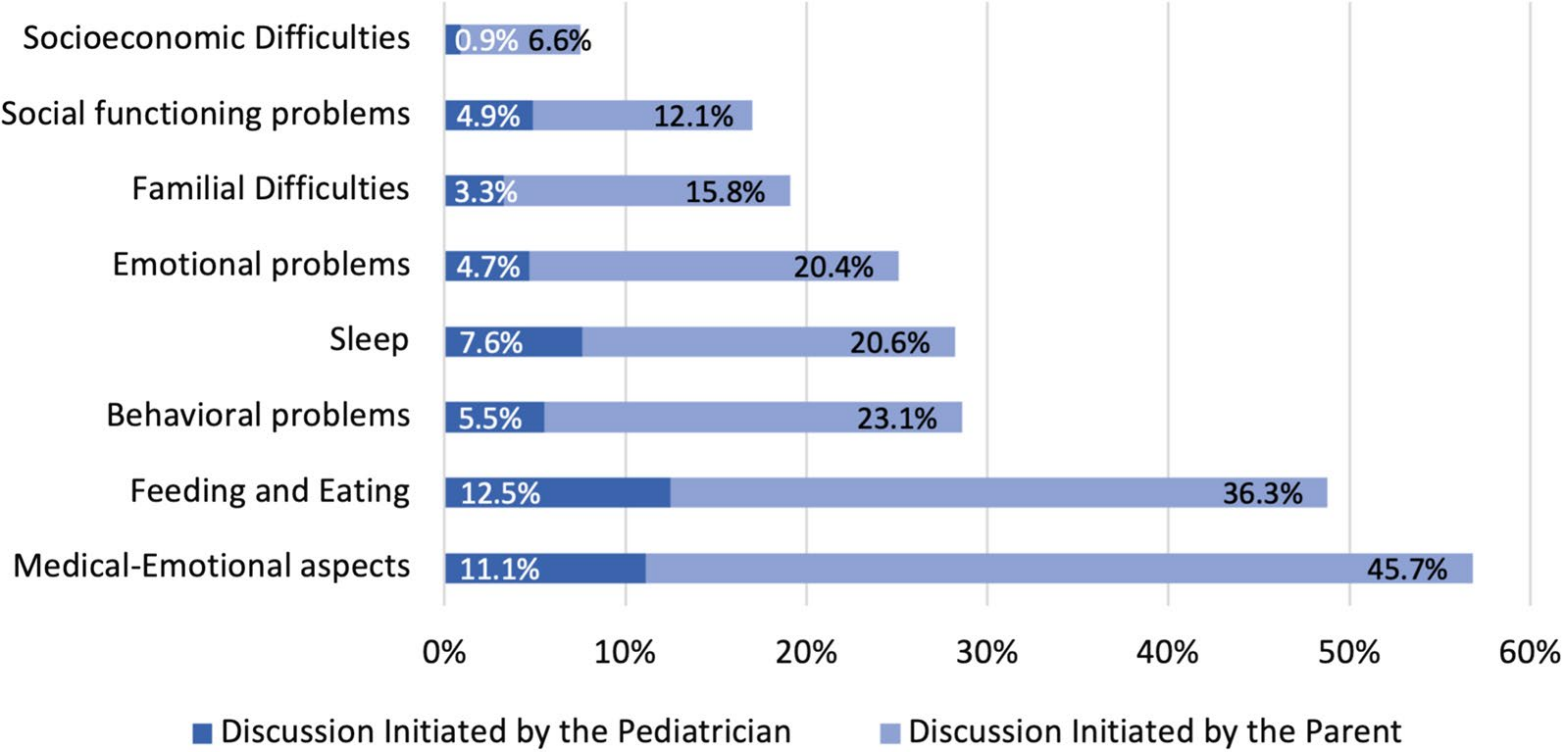
Discussing psychosocial problems with the pediatrician–parent report

For the majority of the problems examined, less than 30% of the parents who expressed a concern regarding one or more of the issues, discussed their concerns with their pediatrician (Fig. 2). Two issues with prominent physical and medical components were exceptional (feeding and eating and emotional aspects of medical conditions), but even these subjects were broached by less than 50% of the parents. Overall, most of the discussions regarding psychosocial issues were initiated by parents.

#### Pediatrician's management of psychosocial problems

Among the parents who expressed concern regarding one or more of the issues (*n* = 549), only 22.3% reported that they received any care from the pediatrician. 21.1% of parents were not satisfied with the care provided by the pediatrician for psychosocial concerns expressed by them. Sixty-four percent of the parents who discussed their concern with the pediatrician, reported that pediatricians had offered treatment options (such as consultation, pharmacological care, or other interventions provided directly by the pediatricians) while 55% reported that they were referred to other professionals. Parents reported that pediatricians tended to refer to other professionals for emotional (73.2%), behavioral (69.6%), or social (64.8%) problems and for family (59.4%) or socioeconomic difficulties (53.5%).

#### Parental barriers

**Fig. 3:**
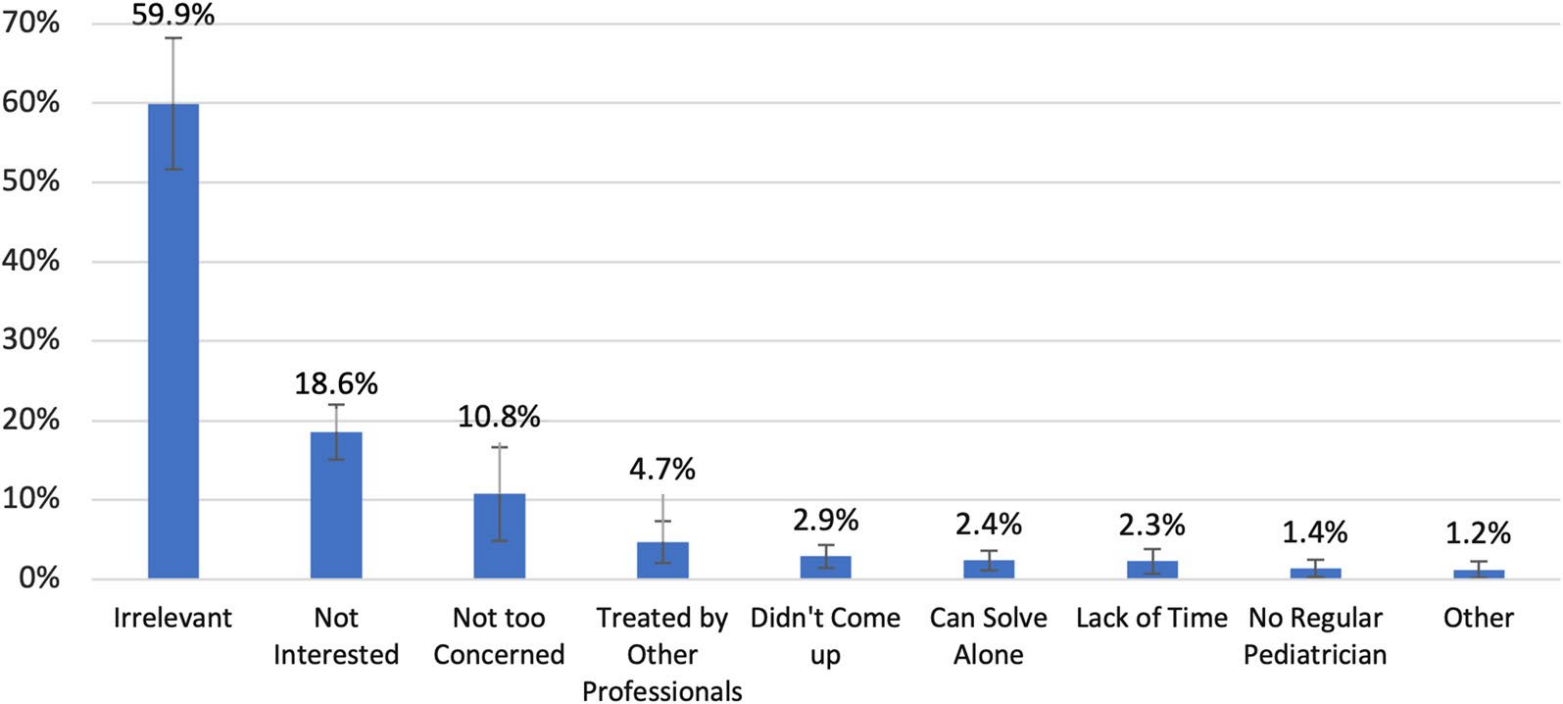
Parents’ barriers

Parents stated several barriers that prevented discussing psycho-social problems with the pediatricians (Fig. 3). The main barrier mentioned by 59.9% of the parents was that psychosocial problems were perceived as irrelevant to the pediatric role. Other barriers were parents’ reluctance to discuss those issues with the pediatrician (18.6%) and a low level of concern (10.8%). Only 2.3% of the parents cited lack of time as a barrier preventing them from discussing psychosocial problems in primary care settings.

### Pediatricians' survey

Demographic Characteristics of the pediatricians are presented in Table 2.

**Table 2:** Demographic characteristics—pediatricians

Variable	Total N = 173
Sex^a^ (*n*, %)	
Male	79 (46%)
Female	91 (54%)
Age (Mean (SD), Range)	55.41 (10.35), 34–82
Years in Practice (Mean (SD), Range)	25.36 (11.98), 0–50
Training in relevant fields^a,b^ (*n*, %)	87 (51.17%)

^a^3 pediatricians did not provide a response to the question

^b^Training includes mental health rotation, community pediatrics or child development clinics rotation, or continuing education in mental health

#### Current practice regarding psychosocial problems

**Fig. 4:**
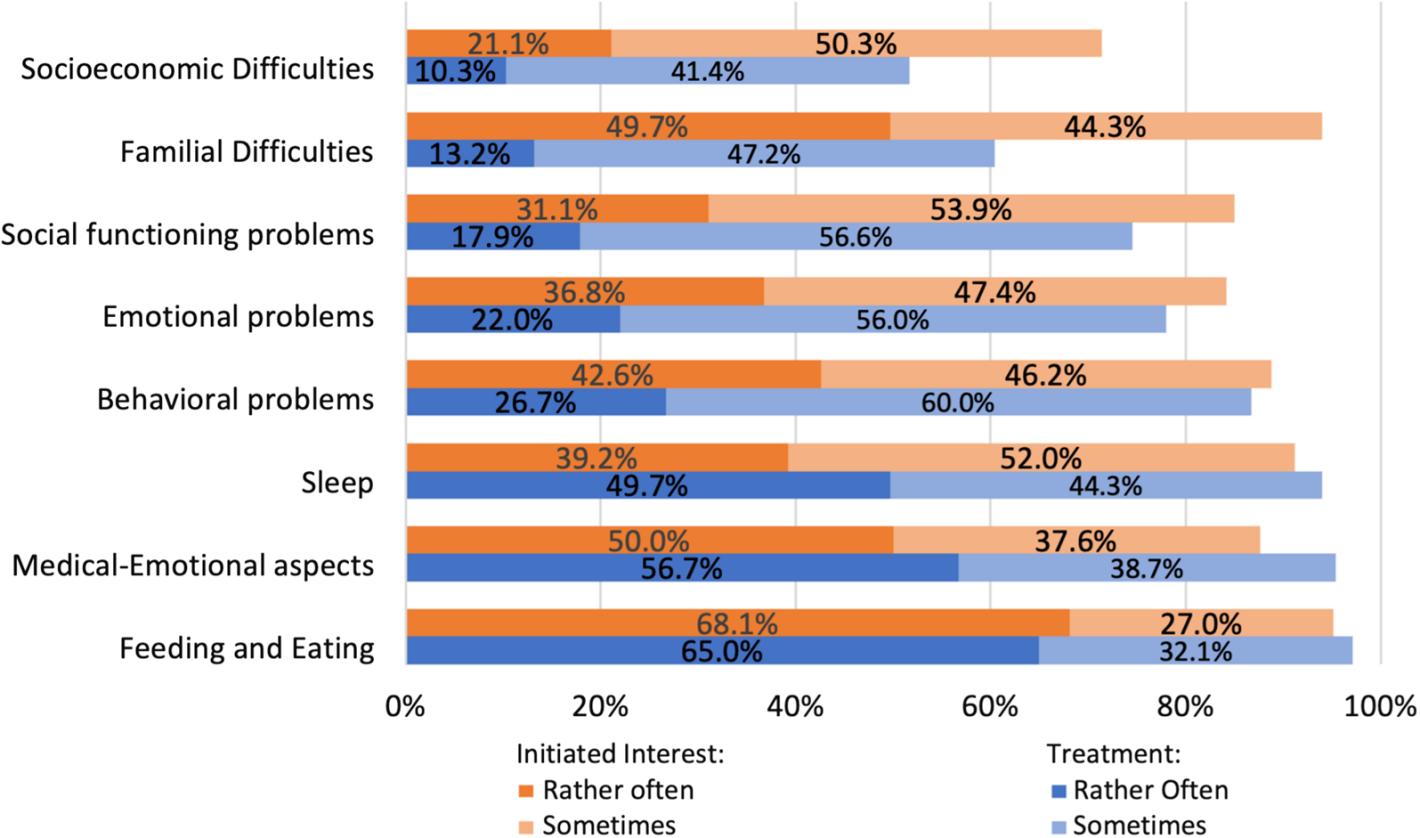
Pediatricians’ practice in psychosocial problems

Pediatricians reporting of initiating inquiries regarding psychosocial issues are shown in (Fig. 4). Socioeconomic difficulties were least discussed by pediatricians (71.4%). Treatment was offered by most pediatricians for problems relating to feeding and eating, sleep, and emotional aspects of medical conditions.

The survey revealed that over the last year, 86.1% of the pediatricians reported that they had referred a patient to a psychologist, 82.7% to a psychiatrist, 78% to other therapists (drama, art, movement, or animal-assisted therapy), and 45.7% to a social worker. 47.9% of the pediatricians reported receiving feedback and maintaining contact with the professional they referred to.

#### Pediatricians' perceived barriers

To further explore the perceived pediatric barriers and due to the multiple correlations between them, an exploratory factor analysis was performed, using the Principal Component Analysis (PCA) method. It was found that the barriers consist of three main factors (eigen values > 1) Pediatricians' lack of professional confidence (including lack of training, knowledge, and confidence to diagnose, consult, or initiate pharmaceutical treatment), Lack of available resources (including lack of time, an inadequate compensation system, and lack of professional services in the community), and Barriers that stem from the parents' perception of psychosocial problems as being irrelevant to the pediatric role. These three factors explained 46.2%, 17.1%, and 13.4% of the variance, respectively. Three variables were calculated by averaging the items loaded on each of these factors.

**Fig. 5:**
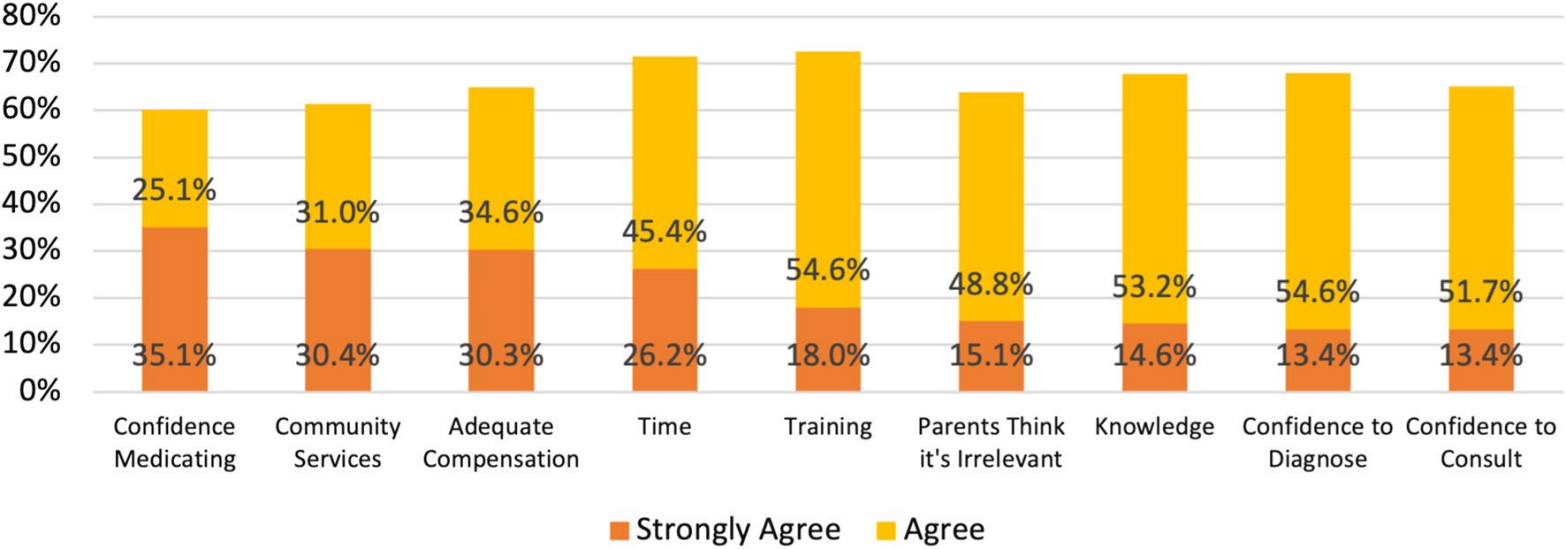
Pediatricians' barriers

The pediatricians’ perception of barriers that prevent them from becoming involved in psychosocial problems are shown in Fig. 5. Lack of time and appropriate training were described as the predominant barriers (71.6% and 72.6%, respectively). Other important barriers raised included lack of confidence regarding pharmaceutical care (60.2%), lack of services in the community (61.4%), and an inadequate compensation system (64.9%).

#### Association between barriers and the pediatricians' practice

The association between the three practice variables (interest, treatment and referral) and the three barriers subgroups (lack of professional confidence, lack of resources and parents' perception of the pediatricians' role) were examined using Pearson correlation coefficients (Table 3). The fewer barriers pediatricians reported regarding lack of professional confidence and resources, the more they tended to initiate conversations about psychosocial problems. A weak negative correlation was also found between a lack of professional confidence and the tendency to refer to other professionals.

**Table 3:** Descriptive statistics and correlations between the study’s variables

	M	SD	1	2	3	4	5
1. Initiated interest	2.25	0.49	1				
2. Treatment	2.13	0.43	.566**	1			
3. Referral	2.28	0.39	.240**	.014	1		
4. Barriers—confidence	3.14	1.04	− .203**	− .016	− .162*	1	
5. Barriers—resources	3.25	1.10	− .227**	− .113	− .155	.438**	1
6. Barriers—parents	3.08	1.24	.114	− .001	.044	− .085	− .072

^*^p < .05; **p < .01

#### The effect of training in relevant fields

Pediatricians with and without training in the relevant fields were compared, using a series of *t*-tests for independent variables, in terms of their interest in psychosocial problems, tendency to treat these problems, tendency to refer to other professionals, and barriers to their involvement (Table 4). Trained pediatricians experienced significantly more barriers related to professional confidence and resources, while untrained pediatricians more often cited as a barrier the parents' view of their role as irrelevant to psychosocial problems.

**Table 4:** Response and barriers of pediatricians with and without training in mental health

	With training	Without training			
	M (SD)	M (SD)	*t*	*p*	Effect size
Initiated interest	2.32 (0.48)	2.19 (0.51)	1.76	.081	
Treatment	2.17 (0.41)	2.07 (0.44)	1.38	.169	
Referral	2.26 (0.36)	2.30 (0.41)	− 0.76	.451	
Barriers—confidence	3.30 (0.93)	2.97 (1.13)	2.10	.037*	*d* = 0.32
Barriers—resources	3.43 (1.04)	3.04 (1.12)	2.32	.022*	*d* = 0.36
Barriers—parents	2.89 (1.32)	3.29 (1.14)	− 2.13	.035*	*d* = 0.32

^*^p < .05

## Discussion

The study demonstrated the current perception of primary care pediatricians’ roles in the management of children's psychosocial problems in Israel, from both the parents’ and the pediatricians' perspectives. A major limitation of this research is related to the representativeness of the sample. The low response rate on the parents' survey resulted in overrepresentation of married and highly educated parents, and underrepresentation of parents with primary or secondary education, which could bias the results. Nevertheless, lower proportion of divorced or widowed parents in the sample is expected, as it included only parents to children under 10, while the general population data included parents to children up to 17. In the pediatricians' sample the demographic characteristics were quite similar to those of all Israeli pediatricians, yet the recruitment of participants through a professional conference and the IPRONET mailing list might have led to an overrepresentation of continuing-education and research-oriented pediatricians and bias the results. In addition, only pediatricians participated in the current study, while 25% of children in Israel are treated by family doctors, who are far more equipped for psychosocial problems, due to their community training and educational program [21]. Thus, some of the parents in the survey receive service from physicians with different training, which might account for at least some of the discrepancies between the parents' and the pediatricians' reports on initiated interest in psychosocial problems. Another limitation concerns the lack of information regarding the differences between the participants and the parents or pediatricians who refused to participate in the study. Thus, the results of this study should be interpreted with caution until reproduced with a more representative sample.

Although approximately half of the parents surveyed were concerned about psychosocial problems, the majority reported that they do not discuss these issues with their pediatrician. The major reason for parents avoiding discussing their concerns with pediatricians was their perception that these concerns were not relevant to the pediatrician's role. This barrier was supported by the parents feeling that the pediatricians rarely initiated discussions regarding their concerns about their child's psychosocial problems.

The problems that parents tended to raise more often were those that they felt the pediatricians was more likely to be interested in, and that involved physical issues (e.g., feeding and eating and emotional aspects of medical conditions). Studies performed in pediatric primary care settings have revealed that parents tend to discuss psychosocial problems with a pediatrician when the family is experiencing a financial crisis or when the child’s behavior is particularly concerning [22, 23]; in other words, only when they feel it is urgent and that they have no choice. Parents need pediatricians to initiate discussions regarding various psychosocial issues in order to feel that primary care is an appropriate setting for their concerns. Given that there is growing evidence suggesting that pediatricians' involvement in psychosocial issues is crucial for early detection and intervention, pediatricians need to be proactive in raising these issues, as opposed to depending on parental initiation to allow for appropriate discussion.

In contrast to the parents' view, pediatricians reported that they were initiating discussions regarding many of the psychosocial problems presented by families. The probable explanation for this discrepancy is that the pediatricians do ask about psychosocial issues in those families that are known to have problems, and that the pediatricians are unaware of many other families that have these concerns since they have never broached the topic. A study conducted in the USA showed that, on many occasions, due to feeling uncomfortable regarding the topic, pediatricians avoided talking to parents about their children’s psychosocial problems [24]. In Israel a previous study showed that 29% of the pediatricians avoid the management of psychosocial issues [14]. It is likely, considering the parents’ responses, that pediatricians in the current study underestimated the shared avoidance of these issues by both themselves and the parents. This problem has been previously reported in the literature, with the assertion that it often results in many children receiving only pharmaceutical treatment [25]. These findings indicate the need for interventions to raise the pediatricians' awareness regarding the prevalence of untreated psychosocial problems, and the necessity of routinely inquiring despite the parents' perception of their role.

Pediatricians in the current study reported that time, lack of training and professional confidence, and inadequate compensation were barriers that prevent them from becoming more involved in psychosocial problems, in accordance with previous research [26, 27]. These barriers were previously found to be associated with high referral rates in primary care settings [28]. Additional barriers mentioned by the Israeli pediatricians included the lack of services in the community and the parents' perception that their roles are irrelevant to psychosocial problems. Negative associations were found between the pediatricians’ tendency to raise psychosocial issues and the barriers of lack of confidence and lack of resources, confirming that these barriers interfere with the pediatricians’ ability to be more involved in psychosocial problems.

Interestingly, pediatricians who had received some advanced training in mental health or developmental centers reported that professional confidence and lack of available resources were the more substantial barriers. In contrast, pediatricians without such training reported the parental perception of psychosocial problems as being irrelevant to the pediatrician's role as the main barrier. This finding appears to be somewhat counterintuitive, where previous researchers found an association between training in the field of psychosocial problems and physicians’ confidence in their ability to treat these issues [29]. A possible explanation is that pediatricians with no additional training do not fully accept that their roles encompass psychosocial problems. Thus, due to the parents' perception, they are more likely to report that they do not have a mandate to treat psychosocial issues. These pediatricians do not serve as change-agents, as they tend not to engage with the promotion of a current professional role perception that responds to the total health and developmental needs of children. It is presumed that training allows for a deeper understanding of the importance of psychosocial problems in pediatric practice. Trained pediatricians are less likely to be influenced by the parents’ perception and more capable of internal attribution of barriers, such as confidence in their abilities. The fact that the trained pediatricians feel insecure in their ability to diagnose and treat psychosocial problems suggests that the training they underwent raised awareness of their lack of knowledge yet failed to provide them with the required skills and led to a feeling of insufficiency. A previous study conducted in Europe revealed a great variation in the formal training for pediatricians who work in community-based settings, from established curricula to no teaching at all [30]. In Israel, pediatricians are trained mainly in hospital-based settings, where a psychiatric consultant is the address for all psychosocial issues. Unlike the training in family medicine, the curriculum of residency in pediatrics has no formal uniform requirements, so some pediatricians receive absolutely no training under community-setting. Moreover, this research reveals that even pediatricians who underwent a rotation in community-setting felt unequipped for dealing with psychosocial problems. This emphasizes that current training is far from being sufficient for the role of primary care pediatricians. To enable pediatric management of psychosocial problems there is a crucial need for an official standardized comprehensive training program in children's development and mental health. In addition to mental-health education programs, which are rarely sufficient to establish a sense of competency, efficient training should include constant consultation, feedback and learning through ongoing relationships with mental health professionals. [8, 10, 11]

The AAP specified six competencies for the pediatrician to master in order to provide service in the psychosocial field: (1) *patient care*—the ability to identify the patient's risk and resilience factors to evaluate and promote mental health; (2) *medical knowledge*—expanded mental health knowledge, such as being familiar with diagnosis, screening and diagnostic tests, and efficient interventions; (3) *practice-based learning and improvement*—the ability to investigate, develop, and assimilate treatment and to appraise and improve the quality of service; (4) *interpersonal and communication skills*—the ability to communicate with families and other treatment providers, resulting in effective information exchange; (5) *professionalism*—providing professional and ethical treatment, including cultural sensitivity, confidentiality, and awareness of one's limitations; and (6) *system-based practice*—the ability to coordinate and cooperate with all the relevant resources in the community [4]. High quality training programs should aim at developing these six competencies among pediatricians.

Managing psychosocial issues and difficulties in community pediatrics cannot be considered as a voluntary aspect that depends on the good will of the pediatrician, but is rather part of the core understanding of child health, and the importance of those issues on child health and wellbeing. It is this is part of the AAP definition of community pediatrics. We are witnessing in the last decades a shift from pediatrics as a purely healing profession for sick kids, to a more holistic health and wellbeing approach, in several places around the world. This should be reflected along the professional training path of every community pediatrician. This is a continuous process starting from experience as a medical student, through residency training and to Continuous Medical Education through the pediatric career. This pathway must include the bio-psych-social model or the ecological model whenever presenting a patient, with assimilation of relevant psychosocial considerations in clinical presentations and providing professional role models. This is not the sole responsibility of any organization, but rather should be a common goal during training of a new generation of pediatricians in Israel, as well as enriching and empowering practicing pediatricians.

There is a need for a long term paradigm shift, and not a short term program to operate, with slow implementation of change.

In addition to a major revision in pediatric training, the current study revealed the need for more resources (time, monetary compensation, and available resources and services in the community) to adequately treat psychosocial problems. The influence of lack of resources on mental health care has been previously examined in a number of studies [31, 32]. The consequences of short visit times were tested in a comprehensive meta-analysis that found that, during shorter visits, less time was spent on topics related to psychosocial problems [33]. Therefore, the AAP consistently refers to visit length and monetary compensation as means of improving medical treatment of psychosocial problems among children and teens [4]. In the current research, the parents did not report time as a barrier. This might be because the majority of the parents did not consider psychosocial problems as part of the pediatrician's role, and therefore, time was not seen as being relevant. Another possibility is that the pediatrician, not the parent, is responsible for the time limits and their required quota and, therefore, they are the one to recognize this factor as an obstacle. In Israel, the pediatrician-to-patient ratio is 0.8 doctors for every 1000 children [34]. This ratio is comparable to the lowest in Europe [35], leading to a considerable overload of patients in pediatric clinics, which limits time for discussing psychosocial issues.

The parents' opinion regarding the nature of the pediatrician’s response is another important measure in the evaluation of the medical services [20]. This study demonstrates that close to a quarter of the parents were not satisfied with the pediatricians’ response to psychosocial problems raised by them. In Israel, parents are highly satisfied with pediatricians’ management of acute problems, despite the low levels of satisfaction with the quality of the response to psychosocial problems [36]. This also supports the assumption that the parents do not expect pediatricians to be involved with non-physical or non-acute issues.

It was previously found that the parents’ level of satisfaction with the quality of the treatment is affected by several factors, for example, the quality of the communication between the parents and the doctor [37]. Effective communication is essential for a fruitful discussion regarding a psychosocial problem and successful implementation of a treatment plan [38]. In addition, initiated interest and empathy improve cooperation and increase parents’ levels of satisfaction [23]. However, the length of a visit did not affect the parents’ level of satisfaction. [39]

### Policy implications and recommendations

Haggerty has claimed that attempts to intervene in mental health issues require familial and social changes [1]. This study emphasizes that a perceptual change for parents is still required to raise psychosocial concerns in primary care. Pediatricians are important change-agents in the process towards the future of pediatrics. To provide adequate psychosocial support, pediatricians should routinely initiate and address psychosocial problems, even when these issues are not raised by the parent.

This study highlights several major barriers to the pediatricians' ability to intervene in the field of mental health and support this process of change. Above all, high quality standardized training is necessary to promote pediatricians' understanding and awareness to the importance of their role in managing psychosocial problems. This training should guarantee a broad knowledge base of diagnoses and risk factors for disorders, some therapeutic abilities, coordination and cooperation abilities, and interpersonal skills and an up-to-date understanding of evidenced-based interventions to support the pediatricians' sense of competence [40, 41]. As with any medical training, community pediatrics should be taught and learned through practice in community pediatric settings, with close monitoring and supervision. To enhance clinical competence. Establishment of appropriate infrastructures will be needed, including curriculum building, appointment of supervisors and supervisor training, and appropriate financial compensation and mechanisms for training counselors and supervisors.

In addition, education and training should also be offered to practicing pediatricians, in order to promote the required paradigm shift in community pediatrics to include mental health and psycosocial issues. Collaboration with mental health specialists is required for ongoing consultation and support, as well as for bridging the gap between the primary care and other mental health community services.

Finally, this study suggests that more resources, such as time and a strong available set of services in the community, are required to support children's psychosocial care in the primary care setting.

## Conclusion

This study examined parents' and pediatricians' current perception of primary care pediatrician’s role in management of psychosocial issues. Representativeness issues were reported, thus future research should corroborate its conclusions with representative samples of both parents and pediatricians. The results of this study revealed many parents avoid discussing their psychosocial concerns with the pediatricians as they perceive this service as irrelevant to the pediatrician's role. Untrained pediatricians reported this parental perception is preventing them from managing psychosocial problems, while trained pediatricians reported that they tend to avoid these issues due to the lack of professional confidence and the lack of available resources. These findings suggest that current pediatric training in children's mental health does not support pediatricians' ability to manage psychosocial problems. Appropriate training is essential for modifying and improving the pediatrician’s perception of their role in managing psychosocial issues in children.

This study, the first attempting to define the role of the community-based pediatrician in managing psycho-social issues, sent two clear messages for the pediatric community. Firstly, there is a glaring lack of training in these issues during pediatric residency training. An almost totally hospital-based training program can never adequately prepare physicians for a future role in the community. Secondly, this study highlights the issue of perceptions of both sides, the pediatrician and the parent, regarding the pediatric role. The wide range of behavioral-developmental, family, socio-economic and educational issues affecting children demand that the pediatrician be aware of these issues, feel confident at managing them directly or via consultation, and that the parents be aware that the pediatrician can be a source of help with the problem. Our findings should serve as a catalyst for shifting the core training curriculum towards the needs of the pediatrician in the community, and for the health service providers to provide the time, the continuing medical education and reimbursement structure to enable the trained pediatrician to use their acquired expertise in the field.

### Supplementary Information

**Additional file 1: Table S1**. Comparison of parents sample and general parents population.

## Data Availability

The data that support the findings regarding parents in this study are available from the Geocartography Knowledge Group, but restrictions apply to the availability of these data, which were used under license for the current study, and so are not publicly available. Data are however available from the authors upon reasonable request and with permission of the Geocartography Knowledge Group. The pediatricians' datasets analyzed during the current study are available from the corresponding author on reasonable request.

## Appendix A: Parent questionnaire

Role in the family:

1. Father
2. Mother

Number of children in the family: ____.

Number of children between the age of 0–10 years: _______.

Ages of children between the age of 0–10 years: ___________.

(The Interviewer will choose one the children who is between 0–10 years old, randomly).

In the coming questions, we will ask you to refer to your child who is ___ years old.

Does your child have an assigned pediatrician, who know them and their medical history?

1. Yes
2. No

How would you define your child’s general health in the last year?

1. Excellent
2. Very good
3. Good
4. Fine
5. Not Good

Did your child ever get treatment from one or more of the following?

1. Speech therapist ____
2. Occupational therapist ______
3. Physiotherapist _____

In what education setting is your child?

1. Home with a family member
2. Home with a nanny
3. Nursery
4. Day care
5. Pre-K/Kindergarten
6. School
7. Other

In the coming questions we will address different types of difficulties that children may have, such as sleep problems, eating and feeding problems, behavioral problems (at home and in the educational setting), emotional difficulties, social difficulties, or family challenges that may affect children, such as divorce, unemployment and the like.

For each of these issues, we are asking if you are concerned about it in your child, and if so, have you talked to your pediatrician (or family physician) about it.

**Table:**

Are you concerned about the following:	Not at all	To some extant*	Yes*
If the answer for one of the questions is “yes” or “to some extent”, the interviewer would move to the question below marked with *, for each questions separately			
Your child's sleep (example: having trouble falling asleep in the evening, waking up often, etc.)			
Your child's eating or feeding (example: eating too little or too much, picky eating, etc.)			
Emotional aspects of recurrent medical symptoms (example: recurrent headaches or abdominal pain, etc.)			
Your child's behavior—as you experience it at home or as reported to you by the educational staff at the kindergarten / school (for example: violence, refusal, discipline problems, etc.)			
Expression of your child's emotional difficulties (example: anxiety, irritability, stubbornness, shyness, insecurity, etc.)			
The way your child connects and communicates with other children			
social problems related to your family (example: unemployment, economic pressures, and more …)			
problems within the family that may affect your child (separation, divorce, chronic illness of one of the parents ……)			

For each of the issues that the parents stated that they were concerned about the interviewer would ask:

* You indicated that you are worried about ____. Have you talked to your pediatrician about this issue?

1. Yes, the pediatrician asked me about this, on they own initiative.
  >> move to question**
2. Yes, I asked the doctor about this, on my own initiative.
  >> move to question**
3. No, I did not ask.
  >> move to question***
4. I do not remember

** Has the doctor suggested treatment options for this issue?

1. Yes
2. No
3. Do not remember

You mentioned that you are worried about one or more of the issues your child is having. As a parent, have you had your needs addressed in these matters?

1. No.
2. Yes—Did you receive the answer from a medical source?
  - Yes. Explain_______
  - No, from another factor. Explain_______

Has the pediatrician referred you to another professional in this regard?

1. No
2. Yes

If so, to whom?

1. Pediatric neurologist / developmental Pediatrician
2. Psychologist
3. Psychiatrist
4. Social worker
5. Emotional therapy including movement / music / art / animal therapy
6. Other ___________________________

*** Why did you not contact your pediatrician about this? (You can mark more than one answer).

1. I did not think it was relevant to the pediatrician's work
2. The pediatrician does not have time for this
3. I am not interested in talking about this issue with the pediatrician
4. Other __________________

How would you rate the quality of the response you receive from your assigned pediatrician on the topics we detailed in this questionnaire?

1. Excellent
2. Good
3. Reasonable
4. Unsatisfactory

In general, to what extent are you satisfied with the treatment given to your child by your assigned pediatrician?

1. Very satisfied
2. Satisfied
3. Fairly satisfied
4. Very dissatisfied

### Demographic characteristics

Child’s year of birth ___________.

Child’s gender:

1. Male
2. Female

In which health insurance is your child insured?

1. Clalit
2. Maccabi
3. Meuhedet
4. Leumit

Mother’s characteristics:

Age ____.

Origin:

1. Born in Israel
2. Born abroad:_________ Year of immigration: _________

Current marital status:

1. Single
2. Married/living with spouse
3. Divorced
4. Widowed

Education:

1. Elementary
2. High School
3. Professional Certificate (Including Haredi seminar)
4. Undergraduate Student
5. BA
6. MA
7. PhD

Religion.

1. Jewish
2. Muslim
3. Christian
4. Other____________

Religiosity:

1. Secular
2. Traditional
3. Religious
4. Haredi

Father’s characteristics:

Age ____.

Origin:

1. Born in Israel
2. Born abroad:_________ Year of immigration:_________

Current marital status:

1. Single
2. Married/living with spouse
3. Divorced
4. Widowed

Education:

1. Elementary
2. High School
3. Professional Certificate (Including Haredi seminar)
4. Undergraduate Student
5. BA
6. MA
7. PhD

Religion.

1. Jewish
2. Muslim
3. Christian
4. Other ____________

Religiosity:

1. Secular
2. Traditional
3. Religious
4. Haredi

## Appendix B: Pediatrician Questioner

The questionnaire addresses the following areas as pertaining to mental health in children: sleep problems, eating and feeding problems, medical-emotional issues (such as recurrent abdominal pain and headaches), behavioral problems (at home and educational setting), emotional problems, social relationships with other children, and family issues, including unemployment, divorce, etc.

**Table:**

To what extent do you	Show interest and ask about on your own initiative			Respond and treats by yourself			Refer to another professionals		
	Never	sometimes	Often	Never	sometimes	Often	Never	sometimes	Often
Sleeping problems	1	2	3	1	2	3	1	2	3
Eating and feeding problems	1	2	3	1	2	3	1	2	3
Emotional aspects of recurrent medical symptoms (example: recurrent headaches or abdominal pain, etc.)	1	2	3	1	2	3	1	2	3
Behavioral problems (at home or educational setting)	1	2	3	1	2	3	1	2	3
Emotional problems (example: anxiety, irritability, stubbornness, shyness, insecurity, etc.)	1	2	3	1	2	3	1	2	3
Social problems	1	2	3	1	2	3	1	2	3
Social problems in the family (example: unemployment, economic pressures, and more …)	1	2	3	1	2	3	1	2	3
Problems within the family (separation, divorce, chronic illness of one of the parents …)	1	2	3	1	2	3	1	2	3

During the past year, if you referred patients for mental health care, where did you refer to? (You can mark more than one answer)

1. Hospital mental health center
2. Community mental health center
3. HMO professional
4. Private professional

To which professionals do you refer for mental health problems? (You can mark more than one answer)

1. Psychologist
2. Psychiatrist
3. Social worker
4. Emotional therapy, including movement/music/art/animal therapy
5. Other ___________________________

Of those treated, did you receive feedback from the professional to whom you referred?

1. Not at all
2. Rarely
3. Yes, to some extent
4. Yes, always/often

### Barriers to treatment

Which of the following is a barrier for you to treat mental health problems in children?

Please indicate your agreement to the following statements:

**Table:**

	Extremely	Very much	Moderately	Slightly	Not at all
Lack of training in the field of mental health	1	2	3	4	5
I do not feel I have enough **knowledge** in the field of mental health	1	2	3	4	5
I do not feel sufficient **confidence** to **diagnose** mental health problems	1	2	3	4	5
I do not feel **confident** enough to **give appropriate mental health advice**	1	2	3	4	5
I do not feel sufficient **confidence** to **treat medically** in the field of mental health	1	2	3	4	5
I do not have enough **time** in the clinic to engage in mental health	1	2	3	4	5
There is no adequate **reward mechanism** for caring for children with mental health issues	1	2	3	4	5
Parents do not see me as an address for discussing problems in these areas	1	2	3	4	5
Lack of appropriate counseling and treatment services in the community to address these issues	1	2	3	4	5

### Demographic information

Age _____.

Gender:

1. Male
2. Female

Seniority in the profession (years): _____.

Number of visits per week at your clinic: _____.

Socio-demographic cross-section of the treated population:

1. Above average
2. Average
3. Below average

Type of clinic:

1. Private
2. HMO
3. Child Health Center

Main HMO:

1. Clalit
2. Maccabi
3. Meuhedet
4. Leumit

Clinic location:

1. North
2. South
3. Central
4. Jerusalem

Type of locality:

1. City
2. Town
3. Village/kibbutz

Clinic location:

1. Central
2. Peripheral

How would you rate the availability of mental health services for children and youth in your area?

1. Very good
2. Good
3. Fair
4. Not good
5. I don’t know

### Training

Graduate of medical school:

1. In Israel
2. Abroad

Certification in Pediatrics:

1. In Israel
2. Abroad

During your internship, did you rotated in children’s mental health, for a period of three months or more?

1. Yes, for a period of __ months
2. No

During your internship, did you rotate pediatric clinics in the community or child development clinics, for a period of three months or more?

1. Yes
2. No

Have you participated in the last 5 years in continuing education / lectures / conferences/ course whose main topic was in the field of mental health in children?

1. Yes
2. No

## Abbreviations

AAP: American Academy of Pediatrics
MCHC: Maternal Child Health Clinics
CATI: Computer-assisted telephone interviewing
IAPA: Israel Ambulatory Pediatrics Association
IPRONET: Israel Pediatric Research in Office Setting NETwork
IPROS: The Israel Pediatric Research in Office Setting
IMOH: Israel’s Ministry of Health
PHDS: Promoting Healthy Development Survey
PCA: Principal component analysis
