# Supplementary material for: Enhancing the primary care pediatrician's role in managing psychosocial issues: a cross sectional study of pediatricians and parents in Israel
Source: Isr J Health Policy Res. 2022 Aug 4;11:28. doi: 10.1186/s13584-022-00537-6 (PMC9354415; doi:10.1186/s13584-022-00537-6)
Supplement: Supplementary file 1 — Additional file 1: Table S1. Comparison of parents sample and general parents population. [file 13584_2022_537_MOESM1_ESM.docx]

| Variable | Parents Sample^1^ | General Parents Population^2^ | Test |
| --- | --- | --- | --- |
| Age (Mean (SD), Range) | 41 (5.9), 21-60 | 40.3 (9.7), 19-80 | *t*(2696) = 1.96, p = .050 |
| Marital status (*%*) |  |  | χ^2^(3) = 9.45, p = .024* |
| Single | 0.5% | 4.9% |  |
| Married\Relationship | 96.7% | 86.1% |  |
| Divorced | 2.4% | 6.8% |  |
| Widowed | 0.4% | 2.1% |  |
| Education (*%*) |  |  | χ^2^(3) = 9.7, p = .021* |
| Primary or secondary | 22.6% | 39.7% |  |
| Professional diploma | 21.2% | 16.7% |  |
| Bachelors degree | 28.8% | 27.2% |  |
| Masters or PhD | 20.5% | 16.4% |  |
| Religion (*%*) |  |  | χ^2^(3) = 3.72, p = .293 |
| Jewish | 78.9% | 71.4% |  |
| Muslim | 17.9% | 21.5% |  |
| Christian | 1.5% | 2.9% |  |
| Other | 1.5% | 4.1% |  |
| Level of religiosity |  |  | χ^2^(3) = 6.56, p = .087 |
| Secular | 47.1% | 49.8% |  |
| Traditional | 8.9% | 11% |  |
| Orthodox | 17.5% | 21.8% |  |
| Ultra-orthodox | 26.5% | 17.1% |  |

*Note: *=p<.05*

^1^ Including only parents of children under 10, N = 1,000.

^2^ Including parents of children under 17, N = 1,698.
